# Maternal nutrition modulates fetal development by inducing placental efficiency changes in gilts

**DOI:** 10.1186/s12864-017-3601-1

**Published:** 2017-02-28

**Authors:** Long Che, ZhenGuo Yang, MengMeng Xu, ShengYu Xu, LianQiang Che, Yan Lin, ZhengFeng Fang, Bin Feng, Jian Li, DaiWen Chen, De Wu

**Affiliations:** Key Laboratory of Animal Disease-Resistance Nutrition and Feed Science, Ministry of Agriculture, P. R. China, Institute of Animal Nutrition, Sichuan Agricultural University, Chengdu, 611130 China

**Keywords:** Energy level, Gilts, Fetal weight, Placenta, iTRAQ

## Abstract

**Background:**

Intra-uterine growth restriction (IUGR) and fetal overgrowth increase risks to postnatal health. Maternal nutrition is the major intrauterine environmental factor that alters fetal weight. However, the mechanisms underlying the effects of maternal nutrition on fetal development are not entirely clear. We developed a pig model, and using isobaric tags for relative and absolute quantification (iTRAQ), we investigated alterations in the placental proteome of gilts on a normal-energy-intake (Con) and high-energy-intake (HE) diet.

**Results:**

In the Con group, heavy and light fetuses were found at the tubal and cervical ends of the uterus respectively at 90 d of gestation. Moreover, the heavy fetuses had a higher glucose concentration than the light fetuses. However, a higher uniformity was noted in the HE group. Placental promoters between these two positions indicated that 78 and 50 differentially expressed proteins were detected in the Con and HE groups respectively. In the Con group, these proteins were involved in lipid metabolism (HADHA, AACS, CAD), nutrient transport (GLUT, SLC27A1), and energy metabolism (NDUFV1, NDUFV2, ATP5C1). However, in the HE group they mainly participated in transcriptional and translational regulation, and intracellular vesicular transport.

**Conclusions:**

Our findings revealed that maternal nutrition may alter birth weight mainly through the modulation of placental lipid and energy metabolism, which also provides a possible mechanism to explain the higher uniformity of fetal weight in gilts fed a HE diet.

**Electronic supplementary material:**

The online version of this article (doi:10.1186/s12864-017-3601-1) contains supplementary material, which is available to authorized users.

## Background

Maternal nutrition has substantial implications for fetal health, and intrauterine growth restrictions (IUGR) and fetal overgrowth increase risks to postnatal health [1, 2]. The placenta is a unique organ, which supplies nutrients and respiratory gases by a transplacental exchange from mother to fetus [3]. Fetal growth is determined by maternal nutritional and endocrine environments, which are dependent on placental transport functions [4]. Abnormal fetal growth is associated with alterations to placental nutrient transporter activity [5], and these changes may contribute directly to IUGR or fetal overgrowth [6]. Neonates with IUGR have a greater susceptibility to disease because they are physiologically deprived of energy stores [7] and have an ineffective immune system [8]. In contrast, fetal overgrowth in pregnancies results in an increased probability of dystocia and the development of a metabolic syndrome later in life [9]. Thus far, studies have revealed various patterns of gene expression in the proteins involved in placental nutrient transport at varied maternal nutritional levels. For example, the glucose transporter protein and neutral amino acid transporter have been regarded as important nutrient transporters, which regulate nutrient transport and fetal development [9, 10]. However, we lack an appropriate animal model and systematic comparison for investigating the molecular mechanisms causing variations in fetal weight.

Compared with sheep and rodents, pigs are more similar to humans physiologically and genetically [11], and are therefore regarded as an ideal model for the study of clinical nutrition [12]. Some studies with pig models show that, a negative effect of abnormal fetal growth is that it contributes to a greater within-litter variation of piglet birth weight (CV_BW0_) [13]. Interestingly, Kim et al. (2013) summarized that fetal weight increased linearly from the cervix to the utero-tubal junction on days 102 and 112 of gestation [14]. Thus, using a pig model, the study of the efficiency of the placenta, which is located on both sides of the uterine horn, is likely to reflect the underlying mechanisms of fetal development that are relevant to incidences of IUGR and overweight offspring. Neonates with too low or high birth weights have been a common problem in human health, although humans generally belong to the classification of uniparous animals [15].

Most animal studies have demonstrated that maternal dietary intake during gestation influences birth weight [16, 17]. Nevertheless, mechanisms underlying the processes by which maternal nutrition regulates fetal development remain unclear. Therefore, this study explored the molecular basis of variations in neonate weight caused by varying maternal dietary energy intake levels during gestation. Using a pig model, we investigated the related factors using placenta proteome analyses with isobaric tags for relative and absolute quantification (iTRAQ). Our results will offer new insight towards a better understanding of the molecular basis of maternal nutritional regulation of fetal development through the placenta.

## Methods

### Animal management and experimental design

Animal studies were conducted in accordance with the law of animal protection approved by the Agricultural Animal Care and Use Committee of Sichuan Agricultural University. In this experiment, 28 purebred Large White (LW) gilts with an average weight of 135.54 ± 0.66 kg were used. After mating, the gilts were allocated randomly to two experimental groups. Dietary treatments included two dietary energy feeding levels with different concentrations of the soybean oil supplement: 14.23 MJ of DE/kg (HE) and 12.56 MJ of DE/kg (Con) of the recommendatory energy requirements by NRC (2012) (Table 1). Feed intake of gestational gilts was 2.0 kg/d from d 0 to 30, 2.4 kg/d from d 31 to 90 and 3.0 kg/d from d 91 to farrow. All gilts were housed in individual feeding stalls, and water was provided ad libitum.

**Table 1:** Ingredients and nutrient content of diets (as fed basis)

Item	Dietary energy level, MJ of DE/kg	
	Con	HE
Ingredients, %		
Corn	45.00	45.00
Soybean meal	13.60	13.60
Wheat bran	27.80	27.80
Soy oil	4.50	9.10
Wheat fiber	2.54	0
Soybean fiber	1.10	0
Corn fiber	0.96	0
Salt	0.40	0.40
Choline chloride	0.14	0.14
Calcium carbonate	1.24	1.24
Dicalcium phosphate	1.99	1.99
Vitamin premix^a^	0.05	0.05
Mineral premix^b^	0.50	0.50
Lysine	0.10	0.10
Threonine	0.10	0.10
Chemical compositions		
DE, MJ/kg	12.56	14.23
CP, %	13.92	13.49
Fat,%	7.27	11.78
Ca, %	0.96	0.96
Total P, %	0.79	0.79
Lysine, %	0.69	0.69
Threonine, %	0.46	0.46

Con control group (12.56 MJ of DE/kg), HE high energy group (14.23 MJ of DE/kg)

^a^Supplied the following per kilogram of complete diet: 4,000 IU of vitamin A; 3,250 IU of vitamin D3; 16 IU of vitamin E; 5.2 mg of riboflavin; 20 mg of nicotinic acid; 11 mg of pantothenic acid; 0.12 mg of vitamin B12; 0.13 mg of biotin

^b^Supplied the following per kilogram of complete diet: 170 mg of Fe; 17 mg of Cu; 160 mg of Zn; 35 mg of Mn; 0.3 mg of Se; 0.28 mg of I

### Sample collection

The backfat thickness of the gilts was measured (P2: 65 mm off the midline at the last rib). Four gilts were selected randomly from each group to be slaughtered on d 55 and 90 of gestation respectively, following deep anesthesia with Zoletil 50 (Zoletil 50 Vet, Virbac, France), at a dose of 0.1 mg/kg of body weight, administered by intramuscular injection, the remaining gilts were fed until delivery. Following laparotomy, uteri were immediately removed from the gilts and placed on ice. On day 90 of gestation, umbilical venous blood, which was located towards the uterine cervix and utero-tubal junction, was collected from each fetus. All blood samples were centrifuged immediately after collection (3000 × *g* for 15 min at 4 °C). For future analyses, serum samples were collected and stored at −20 °C. The weights of fetuses located in each uterine horn, from the cervix to the utero-tubal junction, were recorded on d 55 and 90 of gestation. The placentas were carefully isolated and weighed from the uterus of each fetus on d 55 and 90 of gestation. On d 90 of gestation, the placental tissue samples, which surrounded the cervix and utero-tubal junction, were frozen rapidly in liquid nitrogen, after rinsing with cold sterile saline, for later experiments.

### Analysis of serum IGF-1 and glucose

Serum IGF-1 and glucose were measured using enzyme-linked immunosorbent assay kits (R&D Systems Inc., Minneapolis, MN, USA), according to the manufacturer’s recommendations. The minimum detectable levels of IGF-1 and glucose were 0.01 ng/mL.

### Protein sample preparation

**Fig. 1:**
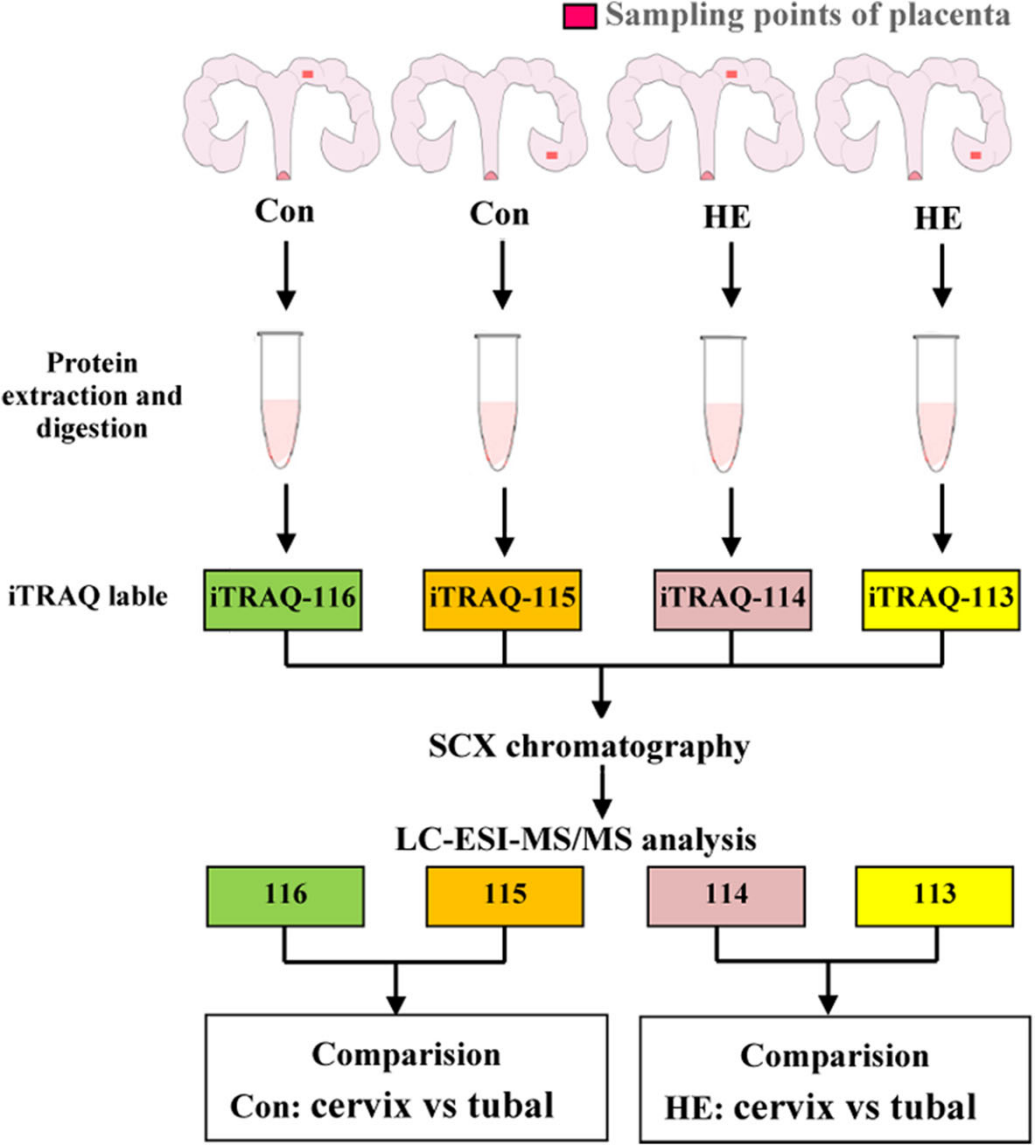
Quantitative iTRAQ proteomics approach. This figure describes the different location of placental sample collection in the uterus between the Con group and the HE group. And the basic process of iTRAQ proteomics approach

The placental samples were obtained from four points on d 90 of gestation: the points at the cervix and utero-tubal junction of the HE group; the points at the cervix and utero-tubal junction of the Con group (Fig. 1). Each point consisted of two biological replicates chosen from four confirmed pregnant gilts in total. Each biological replicate sample was a pooled sample from four randomly selected placental samples, supplied by two gilts at the same uterine site (each gilt provided two placenta samples, one from the right and left of the uterus). Protein was extracted from placental tissues as described previously [18]. The placental samples were ground into powder in liquid nitrogen, extracted with a lysis buffer (7 M urea, 2 M thiourea, 4℅ CHAPS, 40 mM Tris–HCl, pH 8.5) containing 1 mM PMSF and 2 mM EDTA, then 10 mM DTT was added to the samples after 5 min. These solutions were separated by centrifugation at 30,000 × *g* for 15 min at 4 °C. To the supernatant, 10 mM DTT was added to reduce disulfide bonds. Before adding chilled acetone, it was incubated for 1 h in a darkroom using 55 mM IAM to block the cysteines. Subsequently, at 4 °C, samples were centrifuged at 30, 000 × *g* for 15 min. The supernatant from each group was prepared for future analyses. The protein concentrations were determined using a bicinchoninic acid (BCA) protein assay.

### Placenta proteomics assays

The total protein (100 μg) of each sample was digested with Trypsin Gold (Promega, Madison, WI, USA) at a ratio of protein:trypsin = 30:1 at 37 °C, for 16 h. Peptides were then dried and reconstituted in 0.5 M TEAB and processed according to the manufacture’s protocol for the 8-plex iTRAQ reagent. The iTRAQ labeling procedure was as described by previous research [19]. Samples were labeled with the iTRAQ tags as follows: tag 113 for the utero-tubal junction of the HE group, tag 114 for the cervix of the HE group, tag 115 for the utero-tubal junction of the Con group, and tag 116 for the cervix of the Con group. The strong cation exchange (SCX) fractionation, following previous reports [20, 21], was used after the labeled samples were mixed. After being reconstituted with the 4 mL buffer A (25 mM NaH2PO4 in 25% ACN, pH 2.7), the iTRAQ labeled peptide mixtures were briefly loaded onto a 4.6 × 250 mm Ultremex SCX column containing 5-μm particles. The peptides were eluted at a flow rate of 1 mL/min, with a gradient of buffer A for 10 min, 5–60% of buffer B (25 mM NaH2PO4, 1 M KCl in 25% ACN, pH 2.7) for 27 min, and 60–100% of buffer B for 1 min. The fractions were collected every 1 min by measuring the absorbance at 214 nm. Twenty fractions were collected to desalt with a Strata X C18 column (Phenomenex) and vacuum-dry.

### LC-ESI-MS/MS analysis based on Triple TOF 5600

The protocol for MS analysis was as described previously by [22] with slight modifications. Firstly, each fraction was resuspended in buffer A (5% ACN, 0.1% FA) and centrifuged at 20000 × *g* for 10 min, and the final concentration of the peptide was about 0.5 μg/μl. Subsequently, 10 μl of the supernatant was loaded onto a 2 cm C18 trap of the column LC-20 AD nanoHPLC pump system (Shimadzu, Kyoto, Japan). The peptides were eluted onto a 10 cm analytical C18 column (inner diameter 75 μm) packed in-house. The samples were loaded at 8 μL/min for 4 min, then the gradient started at 300 nL/min, from 2 to 35% B (95% ACN, 0.1% FA) for 35 min, linear gradient to 60% for 5 min, 80% for 2 min, maintenance for 4 min, and finally returned to 5% for 1 min. The fractions were analyzed using a TripleTOF 5600 System (AB SCIEX, Concord, ON) with a Nanospray III source (AB SCIEX, Concord, ON) and a pulled quartz tip as the emitter (New Objectives, Woburn, MA). Data were acquired in 250 ms, and as many as 30 product ion scans were collected if a threshold of 120 counts per second (counts/s) was exceeded, with a 2+ to 5+ charge-state and a 15-s dynamic exclusion setting. Each fraction was analyzed by the nano LC-MS/MS as described previously by [23].

### Data processing and analyses

All mass spectrum data were analyzed using Proteome Discover 1.2 (Thermo Fisher Scientific, Bremen, Germany), and the MGF files were searched. Protein identifications were performed using the Mascot search engine (Matrix Science, London, UK; version 2.3.02) against the database Uniprot_pig (Nov 1, 2015). For protein identification, a mass tolerance of 15 ppm was permitted for intact peptide masses, and 20 mmu for MS/MS tolerance and maximum missed cleavages: 1, with allowance for one missed cleavage in the trypsin digests. To reduce the probability of false peptide identification, only those peptides with significant scores (≥20) at the 99% confidence interval by a Mascot probability analysis greater than “identity” were counted as identified [24], in which at least one such unique peptide match was specific for the protein. Gln- > pyro-Glu (N-term Q), Oxidation (M), and Deamidated (NQ) were the potential variable modifications, and Carbamidomethyl (C), iTRAQ8plex (N-term), and iTRAQ8plex (K) were the fixed modifications. The charge states of the peptides were set to +2 and +3. An automatic decoy database search was performed in Mascot by choosing the decoy checkbox in which a random sequence of the database as well as the real database, is generated, and tested for raw spectra. Proteins with a 1.2-fold change or greater, and ratios with *p* < 0.05, were considered significant. The quantitation was performed at the peptide level by following the procedures described in http://www.matrixscience.com/help/quant_statistics_help.html. The student’s *t*-test was performed using the Mascot 2.3.02 software [25]. A protein ratio is reported in bold face if it is significantly different from unity. The comparison test is Student’s t statistic in log space: $\left|\overline{x}\right.-\left.\mu \right|\le t*\frac{s}{\sqrt{N}}$. If this inequality is true, then there is no significant difference at the stated confidence level. (N is the number of peptide ratios, s is the standard deviation and x the mean of the peptide ratios, both numbers calculated in log space. The true value of the ratio, μ, is 0 in log space. t is students t for N-1° of freedom and a two-sided confidence level of 95%).

### MRM validation of differentially expressed proteins from iTRAQ

A spectral library of MS/MS data was generated on a TripleTOF5600 (AB SCIEX, Foster City, CA) and searched against a pig database (48278 entries) using Mascot 2.3.02 (Matrix Science, UK). The date file was imported into Skyline software where a library was built. The peptides were selected for multiple reaction monitoring (MRM) method development according to the following criteria: (1) the peptides with unique sequences in the database; (2) a maximum m/z < 1250 (limitation of quadrupole scan), with a peptide length ranging from 5 to 40 amino acids; (3) the absence of methionine; (4) a carbamidomethyl modification on the cysteine and no variable modifications; (5) and no missed cleavage of trypsin. We initially monitored six transitions per peptide to ensure specificity with the criteria that >5 y-ions with the same elution profile were in the same ratios as the spectral library. The predicted retention time of targeted peptides was observed with an IRT strategy. Pooled peptides were digested and a preliminary SRM assay was used to determine where these proteins were detected.

Samples were digested as described and spiked with 50 fmol of β-galactosidase for data normalization. MRM analyses were performed on a QTRAP5500 mass spectrometer (AB SCIEX, Foster City, CA) equipped with an LC-20 AD nanoHPLC system (Shimadzu, Kyoto, Japan). The Mobile phase consisted of solvent A, 0.1% aqueous formic acid and solvent B, and 98% acetonitrile with 0.1% formic acid. Peptides were separated on a C18 column (0.075× 150 mm column, 3.6 μm) at 300 nL/min, and eluted with a gradient of 5-30% solvent B for 38 min, 30-80% solvent B for 4 min, and maintenance at 80% for 8 min. For the QTRAP5500 mass spectrometer, a spray voltage of 2400 V, nebulizer gas of 23 p.s.i., and dwell time of 10 ms were used. Multiple MRM transitions were monitored using unit resolution in both Q1 and Q3 quadrupoles to maximize specificity. Each MRM transition had a minimum dwell time of 10 ms. We used Skyline 3.5.0.9320 software to integrate the raw file generated by QTRAP 5500 (SCIEX, Framingham, MA, USA) [26]. We used an IRT strategy to define the chromatography of a given peptide against a spectral library. All transitions for each peptide were used for quantitation, unless interference from the matrix was observed. A spike in β-galactosidase was used for label-free data normalization. Data analysis was performed using Skyline. We use an iRT strategy to define a chromotography of a given peptide agianst a spectral library. All transitions for each peptide was used for quantitation unless interference from the matrix was observed. A spiked of β-galactosidase is used for lable free data normalizaiton. We used MSstats 3.4.0 with the linear mixed-effects model [27]. The *p* values were adjusted to control the FDR at a cutoff of 0.05. All proteins with a *p*-value below 0.05 and a fold change larger than 1.5 were considered significant.

### Statistical analysis

The results of the reproductive performance and serum biochemical indices data were analyzed using a Student’s *t*-test in SPSS (v. 19.0 for windows, SPSS; IBM SPSS Company, Chicago, IL, USA). The variations in fetal and piglet birth weight were analyzed following an arcsine square root transformation. Results with *p* < 0.05 were considered significant. These data were shown as means ± standard error of the mean.

## Results

### Reproductive performance of gilts

**Fig. 2:**
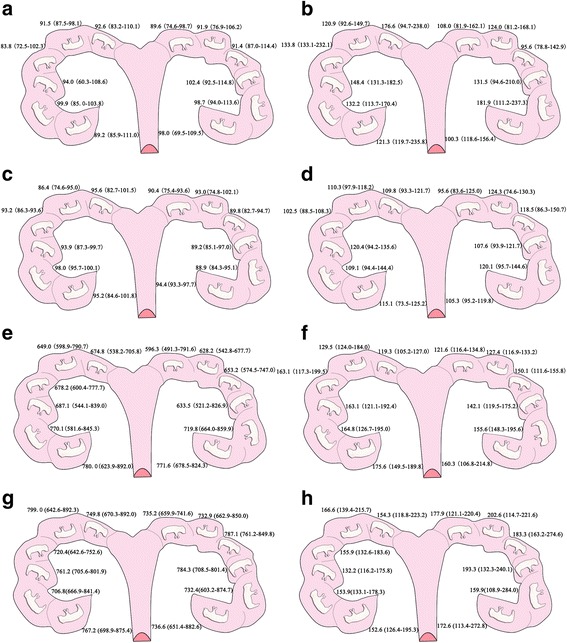
Distribution of fetal and placental weights (**g**) within the uterine horn. **a**: distribution of fetal weights in the Con group on day 55 of gestation; **b**: distribution of placental weights in the Con group on day 55 of gestation; **c**: distribution of fetal weights in the HE group on day 55 of gestation; **d**: distribution of placental weights in the HE group on day 55 of gestation; **e**: distribution of fetal weights in the Con group on day 90 of gestation; **f**: distribution of placental weights in the Con group on day 90 of gestation; **g**: distribution of fetal weights in the HE group on day 90 of gestation; **h**: distribution of placental weights in the HE group on day 90 of gestation. Data are presented as median (interquartile range)

Maternal dietary treatment had no effect on backfat thickness at the P_2_ position, fetal weight, and the coefficient of variation of weight (CV_weight_) on day 55 of gestation (Table 2; *p* > 0.05). However, on day 90 of gestation, the gilts had a higher backfat thickness at the P_2_ position in the HE group than the Con group (*p* < 0.05). Moreover, the fetal weight on day 90 and the piglet weight at parturition were higher in the HE group than the Con group (*p* < 0.05). It is noteworthy that, an increased dietary energy level during gestation influenced the CV_weight_ of the fetus and piglet. The CV_weight_ on day 90 of gestation and farrowing decreased significantly when gilts were fed high energy level diets (*p* < 0.05) (Table 2; Additional file 1: Table S1). The CV_weight_ and distribution did not differ between Con and HE on day 55 of gestation. Interestingly, on day 90 of gestation, the fetal and placental weights of the Con group increased from the cervical to utero-tubal ends of the placenta (Fig. 2e-f); however, no difference in regularity was noted between fetal weight and position within the uterine horn in the HE group.

**Table 2:** Influence of dietary energy level on the reproductive performance of gilts

Items	Days of gestation	Con	HE	*p*-value
Backfat, mm	0	12.33 ± 0.82	12.50 ± 1.05	0.770
	55	15.59 ± 1.03	16.76 ± 1.14	0.240
	90	16.89 ± 0.96	18.65 ± 1.20	0.035
	114	17.17 ± 0.88	20.30 ± 1.21	0.056
Fetal weight, g	55	86.07 ± 3.56	92.59 ± 2.21	0.195
	90	647.18 ± 31.12	780.03 ± 11.81	0.016
	114	1.28 ± 0.11	1.43 ± 0.05	0.012
CV_weight_, %	55	9.8 ± 2.1	7.4 ± 1.9	0.440
	90	15.2 ± 1.2	10.5 ± 1.5	0.047
	114	20.9 ± 2.8	12.0 ± 0.9	0.046

Con control group (12.56 MJ of DE/kg), HE high energy group (14.23 MJ of DE/kg)

CV_weight_: within-litter variation of fetal or piglet. *n* = 4 for each group on days 55 and 90 of gestation; *n* = 6 for each group on day 114 of gestation

### IGF-1 and glucose concentrations in fetal serum at different locations in the uterus

On day 90 of gestation, the IGF-1 and glucose concentrations in the Con group were significantly higher in the fetuses located towards the utero-tubal junction than those towards the cervix (*p* < 0.05). However, no significant differences were found in the HE group (*p* > 0.05) (Table 3; Additional file 2: Table S2).

**Table 3:** Effects of dietary energy level on the serum parameters of the fetuses

Items	Con		*p*-value	HE		*p*-value
	Cervical	Tubal		Cervical	Tubal	
IGF-1 (ng/ml)	14.90 ± 0.55	18.34 ± 0.92	0.018	20.74 ± 1.04	23.69 ± 1.50	0.154
Glucose (ng/ml)	3.89 ± 0.41	5.36 ± 0.18	0.030	4.35 ± 0.75	5.15 ± 0.65	0.464

The serum samples of fetuses were collected on day 90 of gestation. Con: control group (12.56 MJ of DE/kg); HE: high energy group (14.23 MJ of DE/kg). Cervical: the fetuses at the position toward the cervix; Tubal: the fetuses at the position toward the utero-tubal junction. *n* = 8 for each group

### Identification and comparison of proteins of differential abundance

Using iTRAQ analysis, a total of 3011 and 3018 proteins were identified within the false discovery rate of 1% in different uterine locations of the Con and HE groups respectively (Additional file 3: Table S3; Additional file 4: Table S4).

**Fig. 3:**
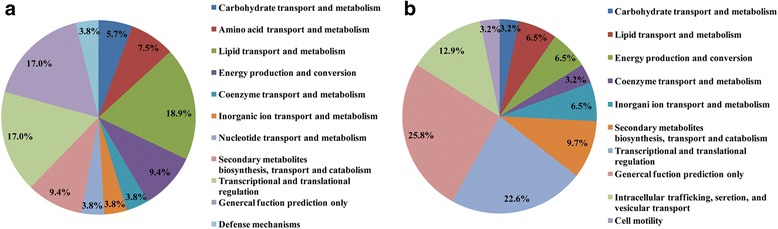
Functional classification of proteins of differential abundance identified from the placenta. Panels show representative figures obtained from **a**: the Con group and **b**: the HE group

Of the identified proteins in the Con group, 78 showed >1.2-fold changes between the cervix and the utero-tubal junction (*p* < 0.05), of which 36 were up-regulated and 42 were down-regulated proteins (Table 4). These differentially expressed proteins were closely involved in lipid, energy, amino acid, nucleotide, coenzyme, and inorganic ion transport and metabolism, as well as signal transduction, cell wall/membrane/envelope biogenesis, posttranslational modification of proteins, defense mechanisms, and miscellaneous. The proteins involved in nutritional transport and metabolism were predominant and accounted for about 62.3% of the differentially expressed proteins. Among these, proteins involved in protein and lipid transport, and metabolism were predominant and accounted for 18.9% (Fig. 3; Additional file 5: Table S5).

**Table 4:** Differentially expressed proteins in the placenta in the Con group

Accession no	Protein name	Unique spectrum	Unique peptide	Gene symbol	Moscot score	Fold change
Carbohydrate transport and metabolism						
gi|262072808	Hexosaminidase B	70	17	HEXB	867	1.28
gi|122134685	Hexokinase-2	26	9	HK2	631	0.79
gi|1956	Glucose transport protein, partial	47	6	GLUT	569	0.79
Lipid transport and metabolism						
gi|350590439	Acyl-CoA synthetase family member 2	19	8	ACSF2	319	1.50
gi|927204434	Acyl-CoA synthetase	2	2	AACS	36	0.70
gi|262072813	17 beta-hydroxysteroid dehydrogenases 12	19	6	HSD17B12	250	0.77
gi|417515459	Solute carrier family 27 (fatty acid transporter) member 4	36	10	SLC27A1	416	0.80
gi|7387634	Mitochondrial trifunctional protein	4	1	HADHA	3812	0.65
gi|113205878	Delta 3, 5, delta 2, 4-dienoyl-CoA isomerase	9	6		169	0.78
gi|640351	Acyl-CoA dehydrogenase	27	9	CAD	527	0.79
gi|356582301	Hydroxymethylglutaryl-CoA synthase	24	8	HMGS	447.5	0.74
gi|350592223	Lanosterol synthase	32	8	LS	475	0.78
gi|27066006	20beta-Hydroxysteroid Dehydrogenase	49	12	20β-HSD	1247	0.70
Amino acid transport and metabolism						
gi|417515548	Selenide, water dikinase 1	13	4	SPS1	262	1.41
gi|75049298	N-acetylneuraminate lyase	68	13	NAL	1427	1.23
gi|927096340	Creatine kinase U-type	50	11	CKMT1	867	0.75
Energy production and conversion						
gi|122138098	Aldehyde dehydrogenase	40	10	ALDH	930	1.35
gi|545835136	NADH dehydrogenase (ubiquinone) flavoprotein 2	48	6	NDUFV2	1107	0.76
gi|311247118	NADH dehydrogenase (ubiquinone) flavoprotein 1	11	6	NDUFV1	111	0.82
gi|343403802	Delta(24)-sterol reductase△24	31	10	DHCR24	461	0.59
gi|753704324	ATP synthase, H+ transporting, gamma polypeptide 1	46	10	ATP5C1	701.5	0.79
Secondary metabolites biosynthesis, transport and catabolism						
gi|545857842	Membrane primary amine oxidase isoform X1	16	7		441	2.63
Coenzyme transport and metabolism						
gi|345110571	Oxygen-dependent coproporphyrinogen-III oxidase	46	14	HemF	822	0.75
gi|545852770	Cytochrome b5 reductase 1	8	5	Cb5R1	106	0.61
Transcriptional and translational regulation						
gi|311260327	Peptidyl-prolyl cis-trans isomerase-like 1	6	2	PPIL1	77	1.25
gi|335308671	60S ribosomal protein L23a	16	6	RPL23A	266	0.78
gi|927177157	40S ribosomal protein S25	26	5	RPS25	271	0.83
gi|927109858	Eukaryotic translation initiation factor 5B	8	5	EIF5B	242	0.77
gi|311276522	ATP-binding cassette sub-family B member 7	6	5	ABCB7	130	0.71

Of the identified proteins in the HE group, 50 showed >1.2-fold changes between the cervix and the utero-tubal junction (*p* < 0.05), where 27 and 23 were up-regulated and down-regulated respectively (Table 5). Although these differentially expressed proteins were also connected with nutrient transport and metabolism, only about 35.6% were involved in, mainly in carbohydrate, coenzyme, lipid and inorganic ion transport and metabolism. The differentially expressed proteins related to lipid transport and metabolism only accounted for 6.5% of the proteins involved (Fig. 3). The other proteins were involved in signal transduction, translation, posttranslational modification of proteins and intracellular trafficking, secretion, and vesicular transport (Additional file 5: Table S5).

**Table 5:** Differentially expressed proteins in the placenta in the HE group

Accession no	Protein name	Unique spectrum	Unique peptide	Gene symbol	Moscot score	Fold change
Carbohydrate transport and metabolism						
gi|937575546	Fucosidase, alpha-L-1, tissue	14	5	FUCA1	163	1.44
Lipid transport and metabolism						
gi|927141698	17-beta-hydroxysteroid dehydrogenase 14	33	8	HSD17B14	657	1.28
gi|927200709	Monoglyceride lipase	14	6	MGLL	319	1.32
Coenzyme transport and metabolism						
gi|281427372	Ferrochelatase, mitochondrial	39	9	FECH	796	1.29
Inorganic ion transport and metabolism						
gi|927135975	N-acetylgalactosamine-6-sulfatase	45	12	GALNS	614	1.50
gi|927207348	Bifunctional 3'-phosphoadenosine 5'-phosphosulfate synthase 2	24	12	PAPSS2	470	0.57
Secondary metabolites biosynthesis, transport and catabolism						
gi|117261	Cholesterol side-chain cleavage enzyme	138	16	SCC	2069.5	1.41
gi|198282077	Cytochrome P450 3A46	26	4	CYP3A46	464.5	1.24
Energy production and conversion						
gi|343432642	Thioredoxin-related transmembrane protein 4	5	3	TMX4	67	1.52
Transcriptional and translational regulation						
gi|545874619	Lupus La protein	38	11		455	1.24
gi|927182150	Sulfhydryl oxidase 1	34	12		454	0.72
gi|927163614	Serpin A3-8	28	6		1264	0.37
Intracellular trafficking, secretion, and vesicular transport						
gi|927170508	Protein transport protein Sec24B	10	4	Sec24B	319	1.40
gi|350596379	Vesicle-trafficking protein SEC22	30	8	SEC22	826	0.80
gi|545802024	Signal peptidase complex catalytic subunit SEC11C	5	2	SEC11C	130	0.70

### GO annotations of proteins of differential abundance

**Fig. 4:**
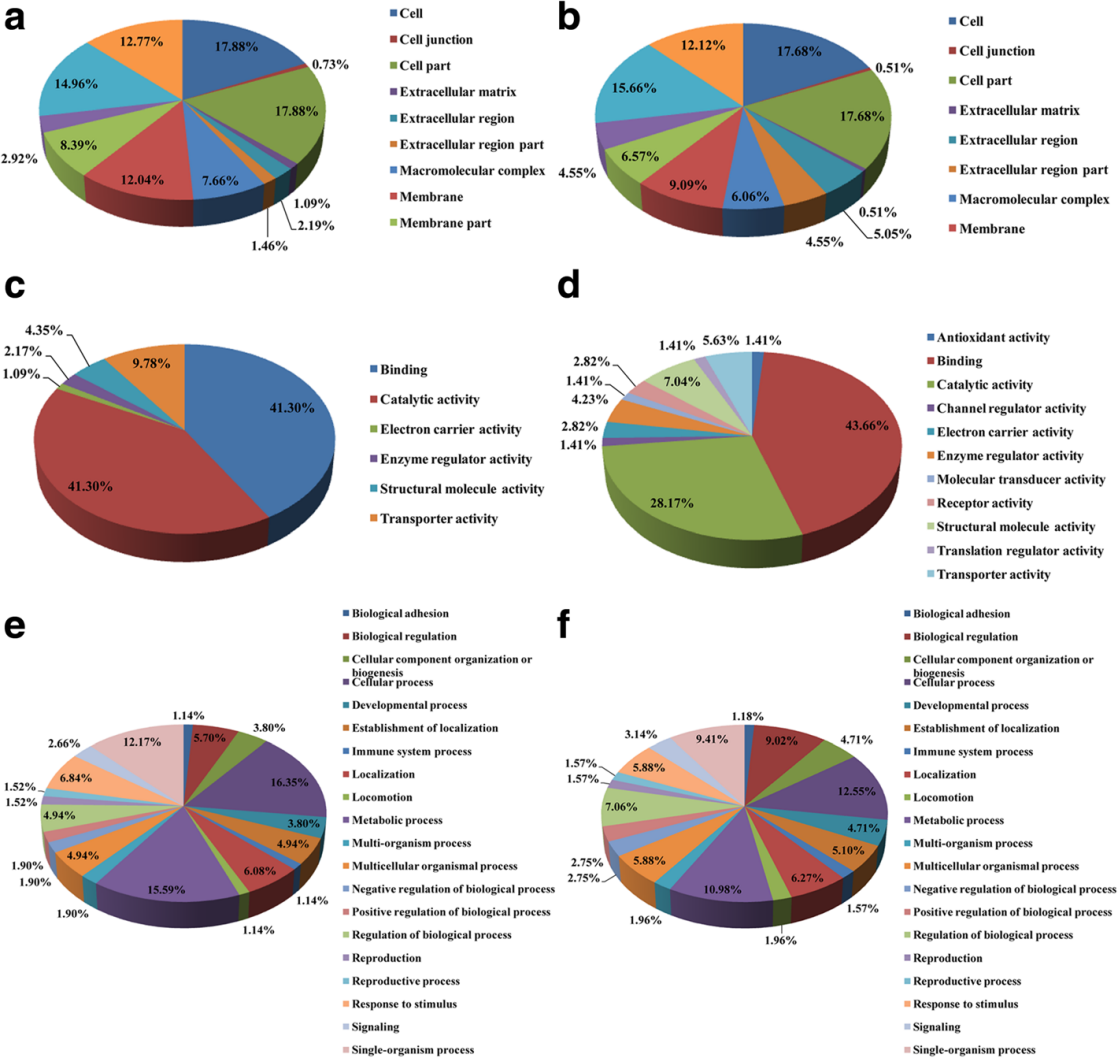
GO distribution analysis of differentially expressed proteins in placenta. **a**: the "cellular component" of Con group; **b**: the "cellular component" of HE group; **c**: the "molecular function" of Con group; **d**: the "molecular function" of HE group; **e**: the "biological process" of Con group; **f**: the "biological process" of HE group

To understand the cellular, molecular, and biological effects of proteins on the development of the placenta between the cervix and the utero-tubal junction in the HE and Con groups, the differentially expressed proteins were categorized according to the Gene Ontology (GO) classes, “cellular component”, “molecular functions”, and “biological process”. Based on the cellular component of the GO analysis, the differentially expressed proteins were concentrated in the intracellular organelles, cytoplasm, membranes, and extracellular regions of both treatment groups. In terms of molecular functions, in the Con group, the differentially expressed proteins that were metabolic enzymes (lyase, oxidoreductase, and catalytic activity) were ranked at the top of the category occupancy, suggesting that nutrient metabolism was predominant in the placenta (Additional file 6: Table S6). However, in the HE group, the proteins were mainly involved in binding (e.g., nucleotide binding, sterol binding, and ion binding). In the biological process category, cellular and metabolic processes were predominant in both treatment groups (Fig. 4). These identified proteins accounted for 16.35% and 15.59% of the cellular and metabolic processes in the Con group respectively, and 12.55% and 10.98% of the cellular and metabolic processes in the HE group respectively, suggesting that an improved maternal dietary energy level affects placental efficiency (Fig. 4). For example, in the Con group, the activities of these proteins mainly included ATP metabolic and biosynthetic processes, and ATP synthesis coupled proton transport, however, in the HE group, the proteins were involved in primarily ion homeostasis and steroid metabolic processes (Additional file 6: Table S6).

### MRM validation for differentially expressed proteins from iTRAQ

**Fig. 5:**
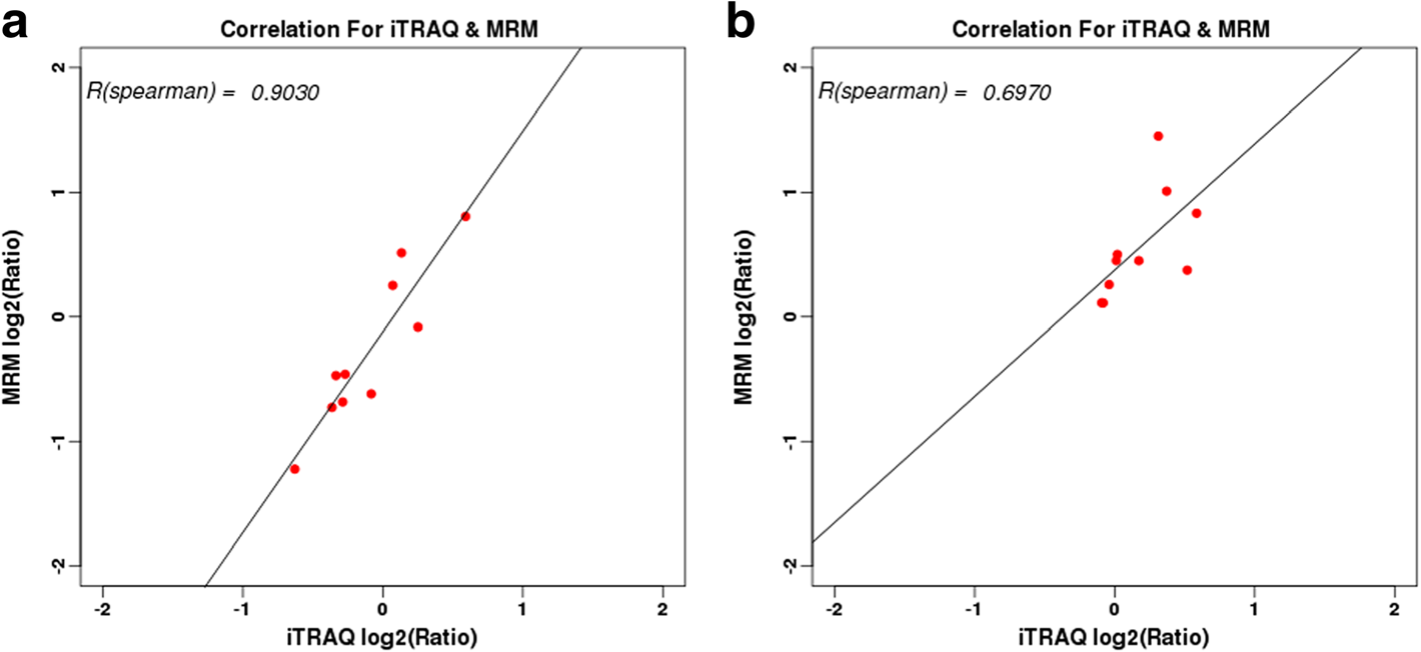
The correlation of fold change between iTRAQ and MRM for the ten target proteins. **a**: the correlation of Con group; **b**: the correlation of HE group

MRM analysis successfully detected 10 differentially expressed proteins (Table 6) including 20 unique peptides in total from iTRAQ. It was important that the log ratios of the quantitative data of the 10 target proteins from MRM were significantly positively correlated with those from iTRAQ (Fig. 5; *p* < 0.01). In the Con group in particular, mitochondrial tri-functional protein and NADH dehydrogenase (ubiquinone) flavoprotein 1 were significantly up-regulated in utero-tubal end of the placenta in iTRAQ (ratio > 1.2, *p* < 0.05) and MRM (*p* < 0.05). These two proteins were involved in lipid transport and metabolism, and energy production and conversion. In the HE group, the results of the two up-regulated proteins in the cervical end of the placenta were consistent in iTRAQ and MRM (*p* < 0.05), and were involved in inorganic ion and coenzyme transport and metabolism. These results further supported the fact that differences in placental efficiency occur between the Con and HE groups.

**Table 6:** MRM validation of differentially expressed proteins in Con and HE groups

Group	Fuction	Protein name	Fold change (iTRAQ)	Fold change (MRM)	*p*-value^*^
Con	Lipid transport and metabolism	Acyl-CoA synthetase family member 2	1.50	1.75	0.05
	Lipid transport and metabolism	Mitochondrial trifunctional protein	0.65	0.43	0.00
	Energy production and conversion	NADH dehydrogenase (ubiquinone) flavoprotein 1	0.82	0.62	0.04
	Energy production and conversion	ATP synthase, H+ transporting, mitochondrial F1 complex	0.79	0.72	0.11
	Transcriptional and translational regulation	60S ribosomal protein L23a	0.78	0.60	0.12
HE	Carbohydrate transport and metabolism	Fucosidase, alpha-L-1, tissue	1.44	1.19	0.39
	Inorganic ion transport and metabolism	N-acetylgalactosamine-6-sulfatase	1.50	1.78	0.01
	Coenzyme transport and metabolism	Ferrochelatase, mitochondrial	1.29	2.01	0.01
	Secondary metabolites	Cytochrome P450 3A46	1.24	2.74	0.19
	Transcriptional and translational regulation	40S ribosomal protein S25	0.83	0.73	0.11

MRM analysis succeeded in detecting 10 differentially expressed proteins. This table showed the differentially expressed proteins in iTRAQ in Con and HE groups

*p-value was considered significant in the results of MRM

## Discussion

The placenta is considered a sensor between maternal nutrition and fetal requirements, and nutrient transport and metabolism are the main physiological events in it [5]. Furthermore, fetal growth is the result of genetic potential modulated by the maternal nutrient supply [28]. Previous studies show that changes in placental nutrient transport may contribute to an altered fetal growth trajectory, and CV_BW_ [29]. However, mechanisms underlying how maternal nutrition regulates fetal development are unknown. Using the iTRAQ-based quantitative proteomics approach, our results showed that the expression patterns of the proteomes in the utero-tubal junction and the cervical end of the placenta varied with different levels of dietary energy intake. In the Con and HE groups, 78 and 50 proteins were differentially expressed respectively. In the Con group, differentially expressed proteins were involved primarily in nutrient transport and metabolism (62.3%), especially lipid and energy metabolism (34%). However, in the HE group, 35.6% of the differentially expressed proteins participated in nutrient transport and metabolism. Furthermore, only 16.2% of these proteins were involved in lipid and energy metabolism. These results indicated that an improved dietary energy level may regulated fetal weight by reducing the numbers of differentially expressed, uterine proteins between the cervix and the utero-tubal junction of the placenta, especially the transport and metabolism proteins. We described a novel pig model in which a high energy level diets during gestation resulted in a decreased CV_BW_. Similarly, Kim et al. (2013) demonstrated that in sows with a limited nutrient supply to support the growth of their fetuses, increased variations in fetal weight during late gestation occurred [14]. To obtain more insight into the mechanisms linking the varied proteomes at different uterine locations to the within-litter birth weight variation, analyses of the differentially expressed proteins of both treatments were carried out.

### Differential proteome study between the placenta toward the position of cervix and utero-tubal junction in Con group

In the Con group, our results showed that fetal weight increased from the cervix to the utero-tubal junction. These results may have at least uncovered the difference in the efficiency of the placental nutrient supply between both locations of the uterus. The positive correlation between the weight of the placenta and birth weight remains a cornerstone of prenatal programming [30]. This study showed that placental weight was greatest at the utero-tubal end, and declined towards the cervical end of the uterus and are consistent with those of Wise et al. (1997). The nutrient transport and metabolism of the placenta was an important determinant of fetal weight. Research has shown that the glucose is the primary energetic material of the feto-placental tissue [31]; however, it originates from maternal lipids stored earlier as an oxidative substrate late in gestation and contributes to a reduction in maternal demand for glucose [32]. Fatty acids are quite essential for the exponential growth of fetuses in the later periods of pregnancy, thus the fetus depends on the maternal diet as well as placental transport and metabolism [33]. Some studies have indicated that in the human fetus, the amount of lipid deposition significantly increases during the third trimester [34, 35]. Therefore, placental transport and metabolism of lipids may be crucial factors determining fetal weight.

In this study, 17 differentially expressed proteins were involved in the transport and metabolism of carbohydrates, proteins, and lipids. First, interestingly, 10 of these abundant proteins were involved in lipids transport and metabolism. The long chain fatty acids (LCFA) that are transported from the maternal plasma are crucial for fetal growth and development because LCFA synthesis in the fetus is minimal [36]. Fatty acid transport proteins (SLC27A1) are required in biological membranes, to promote the cellular uptake of LCFA and meet the increased nutrient demands of the fetus [37]. The initiation of the oxidation of lipids other than glucose is of critical importance as it decreases the placental utilization of glucose [25]. Mitochondrial tri-functional protein (HADHA) is the key enzyme that catalyzes the final three steps of mitochondrial LCFA β-oxidation, which is the major source of energy for the placenta [38]. The expression of HADHA from iTRAQ was further confirmed by MRM, suggesting it may play a role in the lipid transport and metabolism of placenta. Acyl-CoA dehydrogenase (CAD) is one of the important enzymes participating in protein biosynthesis. Recent studies showed that CAD deficiency in the placenta may produce toxic intermediates of fatty acid catabolism, which lead to a reduction in placental antioxidants and cause endothelial damage [39]. Therefore, the increased expression of SLC27A1, HADHA, and CAD in the utero-tubal end of the placenta is probably indicative of an enhanced placental lipids transport and metabolism mechanism and increased transport of glucose to the fetus. However, it is worth mentioning that the protein acyl-CoA synthetase family member 2 (ACSF2) was expressed at a higher level in the placenta at the cervix than in the utero-tubal junction probably because the fatty acids are taken up from the extracellular medium and cytosolic lipid droplets (LDs) are synthesized by ACSF [40]. Thus, we can speculate that there are more fatty acids at the cervical end of the placenta, which form LDs and decrease the supply of fuel and transfer to the fetus. Second, in the utero-tubal end of the placenta, we found that the glucose transport protein (GLUT) had a higher expression than in the cervix. The glucose transporter family mediates the transport of glucose across the plasma membrane of the placenta. Within the glucose transporter family, GLUT1 and GLUT3 are the primary glucose transporter isoforms expressed in the placenta [41]. Previous studies have shown that a high-fat diet supplementation in the mother caused an up-regulation of placental GLUT1, resulting in fetal overgrowth [9].

Mitochondria are regarded as the “powerhouses” of the cell, generating ATP via oxidative phosphorylation by five different complex enzymes. In the process, the first enzyme of the respiratory chain, complex I contains more than 40 subunits encoded by either mitochondrial DNA or the nuclear genome [42]. Mitochondrial complexes NADH dehydrogenase (ubiquinone) flavoprotein1, (NDUFV 1), and NADH dehydrogenase (ubiquinone) flavoprotein 2, (NDUFV 2) are two important subunits involved in oxidative stress and the production of ATP [43]. It is noteworthy that the expression of NDUFV 1 was consistent in iTRAQ and MRM. Bénit et al. (2003) confirmed that a decrease in the protein content of NDUFV2 led to a decrease in complex I activity [44]. An increase in these proteins in the utero-tubal junction of the placenta could enhance anti-oxidative reactions and promote the generation of ATP. Fourth, amino acid also play a crucial role in the regulation of fetal growth, and a reduced fetal plasma concentration of a number of amino acids are associated with the retardation of intrauterine growth [45]. We found that some down-regulated proteins were related to transcriptional and translational regulation in the cervical end of the placenta, including eukaryotic translation initiation factor 5B (EIF5B), ATP-binding cassette sub-family B member 7 (ABCB7), 60S ribosomal protein L23 (RPL23), and 40S ribosomal protein S25 (RPS25), which indicates a decreased capacity for protein synthesis to maintain placental function and integrity [46–49]. Collectively, the placenta at the utero-tubal junction enhanced the transport and metabolism of carbohydrates, lipids, and proteins and possibly promoted energy production and nutrients transport from the mother to the fetus.

### Differential proteome study between the placenta at the position of cervix and utero-tubal junction in HE group

Interestingly, our results showed that fetal weight from the utero-tubal to the cervical end of the placenta did not vary. Moreover, placental weight did not vary with an improved level of dietary energy. What is important is that there was a lower CV_BW0_ in gilts fed on the high energy supplemented diet. These findings indicate that maternal nutritional status affects placental efficiency of nutrient transport. Previous report showed that supplementing sow diets with high energy levels during the late stages of gestation improved fetal growth [16, 17]. In our iTRAQ data of the HE group, we only detected three differential expressed proteins involved in the transport and metabolism of carbohydrates and lipids, not amino. Therefore, the efficiency of placental transport and metabolism plays an important role in regulating fetal weight. Notably, these proteins were all up-regulated in the cervix, including fucosidase, alpha-L-1 (FUCA1), 17-beta-hydroxysteroid dehydrogenase 14 (HSD17B14), and monoglyceride lipase (MGLL). FUCA1 is one of the most important proteins involved in fucose metabolism. Large increases in mRNA levels of FUCA1 are necessary for the prevention of fucosidosis [50]. HSD17B14 mainly participates in oxidizing both estradiol and testosterone into their less bioactive steroid metabolites, estrone and androstenedione respectively [51]. MGLL is expressed in most cell types and is considered the rate-limiting enzyme in the degradation of monoacylglycerols [52]. Increased levels of these proteins in our study, suggested that enhanced carbohydrate and lipids metabolism along with the production of energy was promoted. In addition to carbohydrate and lipid metabolism, more differentially expressed proteins, primarily related to placental health were more up-regulation in the cervix than in the utero-tubal junction. An example of some of these proteins were cytochrome P450 3A46 (CYP3A46), N-acetylgalactosamine-6-sulfatase (GALNS), and ferrochelatase (FECH). Cytochrome P450 (CYP) proteins are a large family of enzymes that play important roles in the oxidative metabolism of xenobiotics. Within this family, CYP3A46 is a key enzyme in the metabolism of xenobiotics, and is functionally related to the human enzyme, CYP3A4 [53]. Previous studies indicate that CYP3A4 is mainly expressed in the liver and small intestine, and is responsible for the metabolism of approximately 50% of currently used drugs [54]. GALNS are enzymes that remove sulfate groups from the chondroitin 6-sulfate (C6S), a deficiency in GALNS leads to the accumulation of sulfated glycosaminoglycans, resulting in lysosomal storage diseases [55]. In addition, ferrochelatase (FECH), which can catalyze heme biosynthesis, iron transport, and the insertion of other divalent metal ions into protoporphyrin IX, ensures that only ferrous iron is used as the physiological substrate, thus preventing its potentially damaging oxidative toxicity [56]. Therefore, increased expressions of critical proteins related to transport and metabolism change the placenta nutrients supply, thus promoting fetal growth in the cervix while simultaneously ensuring the health of the placenta. Finally, a smaller birth weight variation was observed in the HE group.

## Conclusion

Our study provides the first evidence of an alteration in the response of the proteomes, at the utero-tubal and cervical end of the placenta, to an increased dietary energy intake in gilts. Placental lipid and energy metabolism, and nutrient transport may be crucial in influencing the weight of fetuses. In addition, this study demonstrated that a high-energy supply during gestation enhanced fetal development, improved the placental nutrient supply, and decreased within-litter birth weight variation, ultimately, increasing the fetal weight and the uniformity of piglet birth weight. These findings provide new insight into the mechanisms via which maternal nutrition regulates fetal growth, and improves our understanding of placental efficiency.

## Additional files

Additional file 1: Table S1. Original file supporting: influence of dietary energy level on the reproductive performance of gilts. (XLS 33 kb)
Additional file 2: Table S2. Original file supporting: effects of dietary energy level on the serum parameters of the fetuses. (XLS 26 kb)
Additional file 3: Table S3. Differentially expressed proteins from iTRAQ experiments in Con group. (XLS 112 kb)
Additional file 4: Table S4. Differentially expressed proteins from iTRAQ experiments in HE group. (XLS 82 kb)
Additional file 5: Table S5. Functional classification of proteins of differential abundance identified from the placenta in the Con and HE group. (XLS 24 kb)
Additional file 6: Table S6. GO distribution analysis of differentially expressed proteins in placenta in Con and HE group. (XLS 63 kb)

## Abbreviations

AACS: Acyl-CoA synthetase
ABCB7: ATP-binding cassette sub-family B member 7
ACSF2: Acyl-CoA synthetase family member 2
ATP5C1: ATP synthase, H+ transporting, mitochondrial F1 complex, gamma polypeptide 1
C6S: Chondroitin 6-sulfate
CAD: Acyl-CoA dehydrogenase
Con: Normal-energy-intake
CV_BW0_: Within-litter variation of piglet birth weight
CYP3A46: Cytochrome P450 3A46
EIF5B: Eukaryotic translation initiation factor 5B
FECH: Ferrochelatase
FUCA1: Fucosidase, alpha-L-1
GALNS: N-acetylgalactosamine-6-sulfatase
GLUT: Glucose transport protein
GO: Gene ontology
HADHA: Mitochondrial trifunctional protein
HE: High-energy-intake
HSD17B14: 17-beta-hydroxysteroid dehydrogenase 14
iTRAQ: Isobaric tags for relative and absolute quantification
IUGR: Intra-uterine growth restriction
LCFA: Long chain fatty acids
MGLL: Monoglyceride lipase
NDUFV1: NADH dehydrogenase (ubiquinone) flavoprotein 1
NDUFV2: NADH dehydrogenase (ubiquinone) flavoprotein 2
RPL23: 60S ribosomal protein L23a
RPS25: 40S ribosomal protein S25
SLC27A1: Solute carrier family 27
